# Dietary supplement recommendations by Saskatchewan chiropractors: results of an online survey

**DOI:** 10.1186/2045-709X-21-11

**Published:** 2013-03-07

**Authors:** Kent Stuber, Paul Bruno, Kevyn Kristmanson, Zara Ali

**Affiliations:** 1 6100 Leslie Street, Toronto, ON M2H 3J1, Canada; 2 3737 Wascana Parkway, Regina, Saskatchewan S4S 0A2, Canada; 3 102 - 12 Cheadle St. West Swift Current, Saskatchewan S9H 0A9, Canada

**Keywords:** Chiropractic, Nutrition, Survey, Dietary supplement

## Abstract

**Background:**

Chiropractors receive training in nutrition during their education, previous surveys have found that chiropractors frequently provide recommendations to patients relating to nutrition and dietary supplement intake. However, it has not been ascertained which specific supplements chiropractors recommend or the types of health conditions for which supplement recommendations are made.

**Objective:**

The purpose of this study was to determine which dietary supplements are most commonly recommended by chiropractors in the province of Saskatchewan,Canada and the health conditions for which supplement recommendations are made.

**Design:**

An online survey of licensed chiropractors practicing in the province of Saskatchewan, Canada was distributed three times following online and in-person notifications of the survey.

**Statistical analyses performed:**

Descriptive statistics were reported, predominantly in the form of means and proportions.

**Results:**

A response rate of 45% was obtained. All of the respondents (100%) indicated providing nutritional advice or counselling to patients, while nearly all (99%) indicated providing dietary supplement recommendations to patients. Respondents estimated that they provide nutritional advice or counselling to 31% of their patients on average, and recommend dietary supplements to an average of 25% of their patients. The most commonly recommended supplements were glucosamine sulfate, multivitamins, vitamin C, vitamin D, calcium, omega-3 fatty acids, and probiotics. The most common reasons to recommend dietary supplements were for “general health and wellness” (82% of respondents), “bone health” (74%), “rheumatologic, arthritic, degenerative, or inflammatory conditions’ (72%), and “acute and/or chronic musculoskeletal conditions” (65%).

**Conclusion:**

The majority of respondents indicated providing nutritional counselling and recommendations for dietary supplements to their patients. Respondents generally recommend a small number of dietary supplements and provide these recommendations and counselling to fewer than half of their patients on average, while tending to focus on conditions most closely related to the scope of practice of chiropractors. The findings of this study may have been limited by selection bias owing to the low response rate and as those who respond to surveys are often more likely to respond positively.

## Background

Use of dietary supplements is quite common throughout North America, with surveys indicating a past year usage prevalence of up to 73% in the general public [1]. The most commonly utilized supplements are multivitamins/multi-minerals, followed by single vitamin or mineral supplements; other supplements (e.g. herbal/botanical) are less commonly utilized [1]. In this manuscript we will define *dietary supplements* as encompassing both *herbal supplements* (i.e. those obtained from a botanical source) and *nutritional supplements* (e.g. vitamin and/or mineral formulations, amino acids, and hormones not derived from a botanical source) [2].

Chiropractors are among the numerous health care professionals who provide nutritional and dietary advice to patients. In the typical North American chiropractic educational program students complete two courses in nutrition, one providing the foundations of biochemistry and nutrition, followed by a clinical nutrition course [3,4]. Some chiropractors will continue to additional specialization in nutrition, obtaining post-graduate certificates, graduate degrees, or diplomate status in clinical nutrition [3,4].

A 2009 survey of chiropractors across the United States indicated that approximately 94% make nutritional or dietary recommendations to patients [5]. This is higher than previous surveys of American chiropractors which have typically shown 80% of chiropractors incorporate nutritional counselling in their practices [4,6]. A 2001 survey [6] indicated that the most commonly recommended nutritional products by chiropractors were vitamins (by 78% of chiropractors), followed by minerals (65%), herbal preparations (54%), and nutrient combinations for specific conditions (54%). Previous surveys of registered dietitians indicate that 74% to 97% recommend dietary supplements, while only 13% recommend herbal-based supplements [7,8]. This is compared with 40% of pharmacists [2], 79% of physicians [9], 82% of nurses [9], 72% of cardiologists, 66% of dermatologists, and 91% of orthopedists who have indicated recommending dietary supplements to patients [10].

We conducted an online survey, the primary purpose of which was to determine which dietary supplements are most commonly recommended by chiropractors in the province of Saskatchewan, Canada. The current survey is unique compared with previous surveys of chiropractors on this topic in that it asked the frequency with which chiropractors recommend numerous specific dietary supplements and the health conditions for which they recommend supplements, in addition to inquiring about professional behaviours and post-graduate training related to nutrition.

## Methods

This study consisted of an online survey conducted in September 2012. The inclusion criteria consisted of all chiropractors who were actively practicing in the province of Saskatchewan and members of the Chiropractors’ Association of Saskatchewan (CAS). The CAS distributed the e-mail messages for the authors so that confidentiality of their members’ contact information was not violated. All qualified participants were contacted up to a total of five times, including four e-mail messages and one in-person invitation. Participants were initially contacted by a pre-notification e-mail message. Five days later, two of the authors (PB and KK) attended the CAS Annual General Meeting and announced that the initial e-mail invitation would be distributed two days after the meeting (seven days after the pre-notification message). Subsequently, two reminder e-mail invitations were distributed at further two week intervals (14 and 28 days after the initial e-mail invitation message).

The survey e-mail invitation and reminder messages contained the objective and purpose of the study (as part of a study information sheet), which then linked interested participants to an informed consent form via one of the authors’ (KS) Survey Monkey™ account. Participants who agreed to be involved in the study and agreed to the terms of the informed consent were asked to click on an “I Agree” button, which then directed them to the rest of the survey. The survey consisted of several questions including those related to: demographic and nutrition educational background, professional behaviours related to nutrition, how often they recommend different dietary supplements, and the types of conditions for which they recommend supplements (see Additional file 1). Respondents answered the survey in complete anonymity. There was no compensation for participation in this survey. Survey results were summarized with descriptive statistics, expressed as either means or proportions of respondents, depending on the question’s structure. Ethics approval was received from the Canadian Memorial Chiropractic College Research Ethics Board.

Prior to commencement of the survey study, a convenience sample of three clinicians at the HK Lee Clinic on the Canadian Memorial Chiropractic College campus were asked to review and complete the questionnaire to ensure appropriate question quality, style, and content and to allow for revisions to be made. Minor changes were made to survey questions based on the feedback, producing the final survey that was accessed by respondents. The data provided by the clinicians is not included in the data reported herein.

## Results

Of the 182 active CAS members who were contacted, 81 participated in the survey (45% response rate). Demographic information for the survey respondents and for all chiropractors registered with the CAS at the time the survey was conducted are presented in Table 1. With regards to these demographic data, the survey respondents appear to be representative of the target population of chiropractors.

**Table 1:** Demographic information for survey respondents (n=81) and for all chiropractors registered with the Chiropractors’ Association of Saskatchewan (CAS) at the time the survey was conducted (n=182)

		**Survey respondents**	**All CAS members**
**Gender**	***Female***	34%	30%
	***Male***	66%	70%
**College of graduation**	***Canadian Memorial***	75%	69%
	***Chiropractic College***		
	***Other college***	25%	31%
**Years in practice**	***0-5 years***	17%	20%
	***6-10 years***	11%	12%
	***11-15 years***	16%	17%
	***16-20 years***	22%	18%
	***21-25 years***	10%	10%
	***26-30 years***	7%	8%
	***31-35 years***	5%	7%
	***≥ 36 years***	11%	8%

The respondents indicated varying levels of continuing education course involvement related to nutrition and dietary supplements: 17% had not completed any such courses, 54% had taken between one and five courses, 12% had taken between six and ten courses, and 16% had taken 11 or more courses. The vast majority (94%) did not have or were not in the process of completing a post-graduate certificate, fellowship, diplomate, or Master’s degree in nutrition, while 5% had a post-graduate certificate in nutrition, and 2.5% had a post-graduate fellowship or diplomate in nutrition (one of the respondents had both a post-graduate certificate and fellowship or diplomate).

All of the respondents (100%) indicated providing nutritional advice or counselling to patients, while nearly all (99%) also indicated providing dietary supplement recommendations to patients. On average, the respondents estimated that they provide nutritional advice or counselling to 31% of their patients, and recommend dietary supplements to 25% of their patients. In addition, respondents estimated that they refer an average of 6% of their patients to medical doctors and 11% of their patients to naturopathic doctors for dietary or nutrition-related concerns. The average estimated percentage of patients referred to registered dieticians, nutritionists, and homeopathic doctors was 3% (each).

Table 2 depicts the number of respondents who indicated recommending different dietary supplements to their patients, as well as the number of respondents who sell those supplements in their clinics. The supplements that were recommended most often (i.e. recommended “almost always or all of the time” or “often” by ≥25% of respondents) were glucosamine sulfate, multi-vitamins, vitamin C, vitamin D, calcium, omega-3 fatty acids, and probiotics.

**Table 2:** Number of survey respondents (n=81) who recommend (almost always or all of the time, often, sometimes, rarely, never) and sell the following dietary supplements

**Dietary supplement**	**Number of respondents who provided a response**	**Number of respondents who recommended the supplement**			**Sold in the respondents’ clinic**
		***“Almost always or all the time” or “Often”***	***“Sometimes”***	***“Rarely” or “Never”***	
Glucosamine sulfate/ other forms of glucosamine	81	30*	35	16	23
Chondroitin sulfate	64	7	16	41	6
Methylsulfonylmethane (MSM)	67	15	18	34	13
White willow bark	61	3	13	45	6
Boswellia	61	6	10	45	6
Bromelain	65	6	21	38	9
Quercetin	61	3	14	44	8
Multi-vitamins	72	27*	28	17	21
Any B vitamins	66	13	29	24	10
Niacin specifically	55	3	11	41	6
Folic acid specifically	57	5	13	39	6
Vitamin B12 specifically	57	7	12	38	6
Vitamin C	61	26*	16	19	15
Vitamin D	73	40*	17	16	18
Vitamin E	62	7	19	36	7
Calcium	71	32*	19	20	18
Chromium	59	5	13	41	7
Iron	61	6	17	38	5
Magnesium	63	18	21	24	7
Potassium	59	3	14	42	5
Selenium	58	3	9	46	4
Zinc	59	5	12	42	5
Co-enzyme Q10	64	8	13	43	11
Omega-3 fatty acids	75	41*	15	19	20
Omega-6 fatty acids	65	13	11	41	13
Garlic pills	57	3	8	46	3
Saw palmetto	62	5	13	44	11
Black cohosh	56	2	6	48	6
St. John’s Wort	53	0	5	48	1
Ginkgo biloba	54	0	7	47	3
Echinacea	53	3	11	39	4
Ginseng	53	2	3	48	2
Creatine	52	0	4	48	4
Protein powders	56	6	8	42	6
Homeopathic formulations	56	5	5	46	5
Probiotics	67	20*	18	29	14

* indicates the supplements that were recommended “almost always or all the time” or “often” by ≥25% of respondents.

When asked for the types of conditions or reasons for which they recommend dietary supplements, the most common response was for “general health and wellness” (82% of respondents), followed by “bone health” (74%), “rheumatologic, arthritic, degenerative, or inflammatory conditions’ (72%), “acute and/or chronic musculoskeletal conditions” (65%), “weight loss or management” (36%), “digestive conditions” (35%), “neurologic conditions” (35%), “reproductive conditions such as menopause or premenstrual symptoms management” (27%), and “skin conditions” (25%). The remaining reasons for recommending dietary supplements were indicated by fewer than 25% of the respondents and included “anti-aging” (22%), “hormone imbalances” (20%), “cardiovascular conditions” (17%), “nutritional cleansing or colon health” (16%), “weight gain” (15%), “endocrine conditions” (14%), and “psychological conditions” (10%).

## Discussion

All of the respondents to this survey indicated that they provide nutritional advice or counselling to patients, and 99% provide dietary supplement recommendations. Similarly, a 2009 survey of 2371 chiropractors across the United States indicated that 94% provided nutritional and dietary recommendations [5]. In a survey of 125 New York chiropractors, Holtzman and Burke found that 80% of respondents utilized nutritional counseling in their practices, and dietary supplement prescription was the most common form of such counselling [4]. Respondents were asked to estimate the percentage of their patients to whom nutritional advice or counselling was provided (31% on average) as well as the percentage of patients who received dietary supplement recommendations (25% on average). These rates are lower than those reported by 74 American chiropractors in a 2001 survey, who provided nutritional counselling to an average of 37% of their patients, and recommended dietary supplements to an average of 50% of their patients [6].

Although dietary supplement recommendation to patients appears to be common amongst Saskatchewan chiropractors, only a small number of dietary supplements are frequently recommended (glucosamine sulfate, multi-vitamins, vitamin C, vitamin D, calcium, omega-3 fatty acids, and probiotics). These results are not substantially different from the findings of Hetherwick et al. that the most commonly recommended nutrient-based dietary supplements by California dietitians were calcium, multi-vitamins with or without minerals, vitamin E, vitamin C, and iron [7]. Their most commonly recommended herbal supplement was Echinacea, a finding supported by Cashman et al. in their survey of Massachusetts dietitians [7,11]. Conversely, nearly half of the current survey respondents indicated that they “rarely” or “never” recommend Echinacea. Interestingly, a recent review indicates that Echinacea may be effective in reducing the duration and severity of a cold [12].

When considering the types of health conditions for which respondents recommend dietary supplements, there was a level of concurrence with the specific supplements being recommended. The most common reason for recommending supplements to patients was for “general health and wellness” (by 82% of respondents), which is reflected by multi-vitamins, vitamin C, and probiotics being among the most recommended supplements. Vitamin C has been found effective in preventing the common cold [12], however there is conflicting and insufficient evidence to date regarding the use of multivitamins [13-15] and probiotics [16,17] for the prevention and treatment of different health conditions. The next most common reason to recommend supplements was for “bone health” (74%), which is reflected by vitamin D and calcium being frequently recommended and well supported in the literature and by clinical practice guidelines [18-20]. Omega-3 fatty acids and glucosamine sulfate were also commonly recommended by respondents, and this may be a reflection of their frequently recommending supplements for “rheumatologic, arthritic, degenerative, or inflammatory conditions” (72%) and “acute and/or chronic musculoskeletal conditions” (65%). However, there is conflicting evidence surrounding the use of both omega-3 fatty acids [21-24] and glucosamine sulfate [25-27] for such conditions. Holtzman and Burke found that the most common conditions for which New York chiropractors provided nutritional counselling were osteoarthritis (by 76% of respondents) and osteoporosis (71%), followed by obesity, diabetes, coronary artery disease, fibromyalgia, and allergies (each at least 50%) [4].

In comparing the current results to other health professions, a recent survey of American dietitians found that the most common reason for recommending supplements by this group was bone health (70%), followed by filling nutrition gaps (69%), overall health and wellness (49%), lowering cholesterol (46%), cardiac health (46%), dietary pattern reasons (for vegetarians or vegans for example, 43%), and digestive health (39%) [8]. Dickinson et al. surveyed 900 physicians (including 300 general practitioners) and 277 nurses, and the three most common reasons these groups recommend dietary supplements to patients were for overall health and wellness (41% of physicians and 62% of nurses), followed by bone health (41% and 58%, respectively) and joint health (37% and 37%) [9]. In a survey of 300 cardiologists, 300 dermatologists, and 300 orthopedists, the most common reasons that each group provided for recommending dietary supplements was reflected in their professional specialty [10]. For example, the cardiologists’ most common reason for recommending dietary supplements was to lower cholesterol (58%), followed by heart health (55%) and maintaining healthy cholesterol (36%) [10]. Dermatologists most frequently recommended dietary supplements for skin, hair, and nails (81%), followed by overall health and wellness (30%) and bone health (25%). Orthopedists most frequently recommended dietary supplements for bone health (75%), followed by joint health (73%) and musculoskeletal pain (53%) [10]. The findings from the current survey reflect the trends observed in these surveys in that “general health and wellness” was the most commonly cited reason for Saskatchewan chiropractors to recommend supplements to patients, followed by “bone health”, “rheumatologic, arthritic, degenerative, or inflammatory conditions”, and “acute and/or chronic musculoskeletal conditions.” It is reasonable to expect chiropractors’ recommendations to parallel those of orthopedists as both groups predominantly treat patients with musculoskeletal and degenerative conditions, and this does appear to be the case. However, chiropractors seem to have a substantially greater tendency to recommend supplements for “general health and wellness” than do orthopedists (82% versus 25%) [10].

### Postgraduate education in nutrition

Smith and Spillman found that their chiropractor respondents had completed 84 postgraduate nutrition hours on average [6], while the majority of respondents to the current survey had taken between one and five postgraduate courses on nutrition and 28% had completed at least six postgraduate nutrition courses. A small minority of respondents (6%) had completed formal postgraduate qualification (certificate, fellowship, diplomate, graduate degree) in nutrition. Holtzman and Burke [4] found that 65% of their chiropractor respondents did not feel that they had received adequate education in nutrition, and 47% of the respondents in Smith and Spillman [6] felt similarly. Although the current survey’s respondents were not asked to rate the perceived adequacy of the nutritional education in their chiropractic training, these previous survey findings may in part explain why so many respondents have augmented their education with postgraduate training in nutrition.

### Referral rates to and from other health professionals

A 2009 survey of chiropractors in the United States indicated that chiropractors refer patients to nutritionists and receive patient referrals from nutritionists at a frequency between “Never” and “Rarely” (<1/month), while patient referrals to and from family practitioners occurred at a frequency between “Rarely” and “Sometimes” (1-3/month) [28]. The findings from the current survey are in line with these, as respondents indicated referring a higher percentage of their patients to medical doctors (6% of their patients on average) and naturopathic doctors (11%) for dietary or nutrition-related concerns than to dietitians or nutritionists (3% each).

### Study strengths and weaknesses

The response rate of this survey was 45%, which is low, but still within the anticipated range for surveys of chiropractors [29]. Previous surveys of chiropractors regarding nutritional and dietary supplement recommendations have obtained response rates varying between 13% and 34%. The current study actually had the highest response rate of chiropractors thus far on this topic, making it difficult to necessarily validate the results of the current survey against those conducted previously [4-6]. Numerous steps were taken to encourage a higher response rate, such as pre-notification, and multiple contacts, both in person and through e-mail messages [29]. Respondents to this survey were practicing chiropractors from only one jurisdiction in Canada, which limits the ability to generalize results to other North American jurisdictions. Additionally, not all of respondents answered all of the survey questions, creating a level of uncertainty for those particular results.

Furthermore, while this survey did inquire about different reasons for respondents to recommend dietary supplements, it did not ask respondents to directly relate these reasons to specific supplements. Additionally, the list of supplements occasionally did not necessarily correspond to those different reasons. For example, none of the listed supplements would be considered to be specifically targeted towards “weight loss or management”, which was mentioned as a reason for recommending supplements by 36% of respondents. In addition, all of the survey respondents indicated providing nutritional advice or counselling to patients, and nearly all respondents provide dietary supplement recommendations to patients. These data lead to a concern of some degree of selection bias where only those who do recommend supplements or provide nutritional advice or counselling chose to complete the survey. As mentioned previously Table 1 indicates that the survey respondents do not appear to be essentially different from the entire population of Saskatchewan chiropractors from a demographic standpoint, thus it is unclear if there is any bias in the results due to non-response.

## Conclusions

The majority of respondents to this survey indicated providing nutritional counselling and recommendations for dietary supplements to patients. They provide these recommendations and counselling to fewer than half of their patients on average and tend to focus on conditions most related to the chiropractic scope of practice, including general health and wellness, along with musculoskeletal and degenerative conditions. Respondents mainly recommend a small number of dietary supplements such as multivitamins, vitamin C, vitamin D, calcium, probiotics, glucosamine sulfate, and omega-3 fatty acids. Most of the responding chiropractors have completed postgraduate courses on nutrition, and generally do not refer a high proportion of patients for nutritional counselling to other health professionals. Future research should be directed towards larger jurisdictions which may be more representative of North American chiropractors and should include qualitative research which elucidates the reasons for different professional behaviours relating to nutrition.

## Competing interests

The authors declare that they have no competing interests.

## Authors’ contributions

KS conceived and designed the study, participated in data collection and analysis, and drafted the manuscript. PB contributed to study design, data collection, data analysis, and revisions of the manuscript. KK contributed to study design, data collection, and revisions of the manuscript. ZA contributed to study design and revisions of the manuscript. All of the authors have given their approval of the final version to be published.

## Supplementary Material

Additional file 1 Online Nutritional Survey. Click here for file
