# Supplementary material for: Dietary supplement recommendations by Saskatchewan chiropractors: results of an online survey
Source: Chiropr Man Therap. 2013 Mar 7;21:11. doi: 10.1186/2045-709X-21-11 (PMC3599949; doi:10.1186/2045-709X-21-11)
Supplement: Additional file 1 — Online Nutritional Survey. [file 2045-709X-21-11-S1.docx]

**Additional file 1: Online Nutritional Survey.**

1. **How many years have you been in practice?**

- 0-5
- 6-10
- 11-15
- 16-20
- 21-25
- 26-30
- 31-35
- 36+

1. **Where did you graduate from chiropractic school?**

- CMCC
- UQTR
- United States
- Europe
- Australia
- New Zealand
- Other

1. **Please indicate your gender.**

- Male
- Female

1. **Since graduating from chiropractic school, how many continuing education courses on nutrition and/or nutritional supplements have you taken?**

- 0
- 1
- 2
- 3
- 4
- 5
- 6-10
- 11+

1. **Have you completed any expert level certifications, diplomates, fellowships, or graduate degrees specifically on nutrition? (you may indicate more than one response if needed)**

- No I have not completed any of these forms of training
- Yes, certificate in nutrition completed
- Yes, diplomate or fellowship in nutrition completed
- Yes, graduate degree in nutrition completed (Master’s or PhD)
- Yes, I am completing a certificate in nutrition currently
- Yes, I am completing a diplomate or fellowship in nutrition currently
- Yes, I am completing a graduate degree (Master’s or PhD) in nutrition currently

1. **To what percentage of your patients would you estimate you provide the following?**

- Nutritional advice or counseling: __________
- Nutritional supplement encouragement: __________

1. **Please indicate which percentage of your patients you refer to the following health professionals for dietary or nutrition related concerns.**

- Nutritionist: __________
- Registered Dietician: __________
- Medical Doctor: __________
- Naturopathic Doctor: __________
- Homeopathic practitioner: __________

1. **Please indicate the types of reasons or conditions for which you encourage nutritional supplements. (you may indicate as many responses as necessary)**

- General health and wellness
- Anti-aging
- Nutritional cleansing/colon health
- Weight loss or management
- Weight gain
- Musculoskeletal conditions – acute and/or chronic
- Rheumatologic/arthritic/degenerative/inflammatory conditions
- Neurological conditions
- Reproductive conditions (such as menopause or premenstrual symptom management)
- Hormone imbalances
- Skin conditions
- Digestive conditions
- Endocrine conditions
- Cardiovascular conditions
- Psychological conditions
- Bone health (such as osteoporosis or osteopenia)
- Other (please specify): ____________________

1. **For the following nutritional supplements, please indicate how often you encourage that supplement and whether you sell that particular supplement in your clinical practice.**

| **Supplement** | **Encouraged almost always or all the time** | **Encouraged often** | **Encouraged sometimes** | **Encouraged rarely** | **Never** | | **I sell this product in my clinic** |
| --- | --- | --- | --- | --- | --- | --- | --- |
| **Glucosamine sulfate/other forms of glucosamine** |  |  |  |  |  | |  |
| **Chondroitin sulfate** |  |  |  |  |  | |  |
| **Methylsulfonylmethane (MSM)** |  |  |  |  |  | |  |
| **White Willow Bark** |  |  |  |  |  | |  |
| **Boswellia** |  |  |  |  |  | |  |
| **Bromelain** |  |  |  |  |  | |  |
| **Quercetin** |  |  |  |  |  | |  |
| **Multi-vitamins** |  |  |  |  |  | |  |
| **Any B vitamins** |  |  |  |  |  | |  |
| **Niacin (vitamin B3) specifically** |  |  |  |  |  | |  |
| **Folic Acid (Vitamin B9) specifically** |  |  |  |  |  | |  |
| **Vitamin B12 specifically** |  |  |  |  |  | |  |
| **Vitamin C** |  |  |  |  |  | |  |
| **Vitamin D** |  |  |  |  |  | |  |
| **Vitamin E** |  |  |  |  |  | |  |
| **Calcium** |  |  |  |  |  | |  |
| **Chromium** |  |  |  |  |  | |  |
| **Iron** |  |  |  |  |  | |  |
| **Magnesium** |  |  |  |  |  | |  |
| **Potassium** |  |  |  |  |  | |  |
| **Selenium** |  |  |  |  |  | |  |
| **Zinc** |  |  |  |  |  | |  |
| **Co-enzyme Q10** |  |  |  |  |  | |  |
| **Omega-3 fatty acids (fish oils, flax seed oil, chia, etc)** |  |  |  |  | |  |  |
| **Omega-6 fatty acids (Evening Primrose oil, Borage seed oil, etc)** |  |  |  |  | |  |  |
| **Garlic pills** |  |  |  |  | |  |  |
| **Saw palmetto** |  |  |  |  | |  |  |
| **Black cohosh** |  |  |  |  | |  |  |
| **St. John's Wort** |  |  |  |  | |  |  |
| **Ginkgo biloba** |  |  |  |  | |  |  |
| **Echinacea** |  |  |  |  | |  |  |
| **Ginseng** |  |  |  |  | |  |  |
| **Creatine** |  |  |  |  | |  |  |
| **Protein powders** |  |  |  |  | |  |  |
| **Homeopathic formulations** |  |  |  |  | |  |  |
| **Probiotics** |  |  |  |  | |  |  |

- Other (please specify): ____________________
